# Dynamic changes in heparan sulfate during muscle differentiation and ageing regulate myoblast cell fate and FGF2 signalling

**DOI:** 10.1016/j.matbio.2016.07.007

**Published:** 2017-05

**Authors:** R.S. Ghadiali, S.E. Guimond, J.E. Turnbull, A. Pisconti

**Affiliations:** Department of Biochemistry, Centre for Glycobiology, Institute of Integrative Biology, University of Liverpool, Crown Street, Liverpool L69 7ZB, United Kingdom

## Abstract

Satellite cells (SCs) are skeletal muscle stem cells residing quiescent around healthy muscle fibres. In response to injury or disease SCs activate, proliferate and eventually differentiate and fuse to one another to form new muscle fibres, or to existing damaged fibres to repair them. The sulfated polysaccharide heparan sulfate (HS) is a highly variable biomolecule known to play key roles in the regulation of cell fate decisions, though the changes that muscle HS undergoes during SC differentiation are unknown. Here we show that the sulfation levels of HS increase during SC differentiation; more specifically, we observe an increase in 6-O and 2-O-sulfation in N-acetylated disaccharides. Interestingly, a specific increase in 6-O sulfation is also observed in the heparanome of ageing muscle, which we show leads to promotion of FGF2 signalling and satellite cell proliferation, suggesting a role for the heparanome dynamics in age-associated loss of quiescence. Addition of HS mimetics to differentiating SC cultures results in differential effects: an oversulfated HS mimetic increases differentiation and inhibits FGF2 signalling, a known major promoter of SC proliferation and inhibitor of differentiation. In contrast, FGF2 signalling is promoted by an N-acetylated HS mimetic, which inhibits differentiation and promotes SC expansion. We conclude that the heparanome of SCs is dynamically regulated during muscle differentiation and ageing, and that such changes might account for some of the phenotypes and signalling events that are associated with these processes.

## Highlights

• The heparanome of differentiated muscle satellite cells is more highly sulfated than that of proliferating satellite cells.
• Aged muscle heparan sulfate contains higher levels of 6-O-sulfation and promotes FGF2 signalling and satellite cell proliferation
• 6-O-sulfation promotes satellite cell proliferation
• The effect of heparan sulfate on satellite cell proliferation, differentiation and self-renewal is structure- and dose-dependent

## Introduction

Heparan sulfate proteoglycans (HSPGs) are complex biomolecules composed by a core protein to which polysaccharide chains, called heparan sulfate (HS) glycosaminoglycans (GAGs), are covalently bound. HSPGs exert their function through several mechanisms, a major one being the modulation of the bioavailability and activity of heparin-binding growth factors, which depend largely on the structure of the HS GAGs attached to HSPG core proteins [1], [2], [3].

HS is a linear polysaccharide composed by a variable number (10–200) of repeating disaccharide units of N-acetyl- or N-sulfo-D-glucosamine (GlcNAc) linked to an uronic acid, which is either D-glucuronic acid (GlcA) or its epimer L-iduronic acid (IdoA). HS disaccharides can be sulfated in various positions by specific enzymes. The most common positions for HS sulfation are: the 6-O and N- positions of the glucosamine residue and the 2-O position of the uronic acid residue. Additionally, a more rare sulfation occurs on the 3-O position of the glucosamine residue [4]. Heparin is a type of HS that: (i) contains mostly L-iduronic acid instead of D-glucuronic acid in its backbone, (ii) is more highly sulfated (due to a prevalence of N-sulfation as opposed to the prevalence of N-acetylation observed in HS) and (iii) on average is slightly smaller than HS. Although it is typically recognised that HS is produced in virtually all tissues while heparin is produced only by mast cells, it has been shown that HS with features close to those of heparin are also produced by cell types other than mast cells, such as endothelial cells and glia [5], [6]. Moreover, HS with features typical of heparin has been found in various tissues, such as lung and liver [7], [8].

HS plays key roles in multiple biological processes such as cell growth, development, cell signalling and coagulation [1], [9]. The different functions played by HS depend mostly on its structural composition, which arise from the different levels and combinations of sulfation harboured by the disaccharide units [1]. For example, a prototype of HS-protein interaction is the formation of the ternary signalling complex that includes fibroblast growth factor (FGF), FGF receptor and HS. It has been shown that in the formation of this ternary signalling complex specific disaccharides containing specific degrees and patterns of sulfation are preferred over others [10], further supporting the idea that HS has evolved as a highly diverse regulator of cell signalling with binding specificity and selectivity.

Muscle satellite cells (SCs) are undifferentiated, quiescent muscle stem cells residing in a specialised anatomical niche between the muscle fibre plasma membrane and the surrounding basal lamina [11]. In response to injury SCs become activated, proliferate as myoblasts, differentiate and fuse to pre-existing or new muscle fibres thus effectively regenerating the lost muscle fibres [12].

Several GAGs are present in the SC niche, including HS, chondroitin sulfate (CS) and dermatan sulfate (DS) [13], [14]. Interestingly, total sulfated GAG amounts increase in the C2.7 myoblast cell line when myoblasts are differentiated to myotubes [15] indicating GAGs as important regulators of myogenesis – the process of muscle formation. Indeed, expression of various HSPGs is regulated during muscle regeneration and functionally involved in several signalling pathways such as FGF, HGF, Notch1 and Wnt7a [16], [17], [18], [19], [20], [21], [22], [23], [24], [25]. Genetic ablation or modulation of HSPGs expressed in SCs, such as syndecan-3 and syndecan-4, dramatically affect muscle regeneration in response to injury and SC homeostasis [18], [20]. Moreover, genetic ablation of the two extracellular sulfatases that remove 6-O-sulfation from HS, Sulf1 and Sulf2, also leads to impaired SC function [26]. While regulation and function of HSPG core proteins during myogenesis has been studied [27], regulation of HS composition during myogenesis remains a largely unexplored area. Through phage display technology, it has been shown that different HS epitopes exist in skeletal muscle, and their abundance changes in developing muscle *in vitro* and *in vivo* [13], [14]. Moreover, it is known that the amounts of HS and CS increase during ischemia-induced muscle injury and that the levels and patterns of HS and CS sulfation change [28]. These results suggest a potential role for specific structural moieties of the HS chains in regulating skeletal muscle regeneration. However, it is still largely unknown if the structure of SC HS changes during these processes and how different HS structures affect SC function.

During ageing skeletal muscle loses mass and strength, a process called sarcopenia, and regenerative capacity. This is due to both intrinsic SC impairment and changes in molecular signalling that occur in the SC niche [29], [30], [31], [32]. Interestingly, HS regulates several signalling events that are reportedly altered in the aged SC niche, such as FGF2 signalling [31], [33]. Moreover, age-related changes in HS have been reported in some human tissues including aorta and cardiac muscle [34], [35]. Thus, it is plausible that age-associated changes in the muscle heparanome – the complex mixture of various HS structures present in a tissue – underlie some of the molecular mechanisms that impair SC homeostasis and function in aged muscle.

In this study we investigate whether HS sulfation levels and patterns are dynamically regulated during muscle differentiation and ageing and these changes affect satellite cell proliferation and signalling. Moreover, we exploit a library of HS mimetics to systematically test the role of differential HS sulfation in SC differentiation and FGF2 signalling.

## Results and discussion

### The level of sulfation of the muscle stem cell heparanome increases with differentiation

**Fig. 1:**
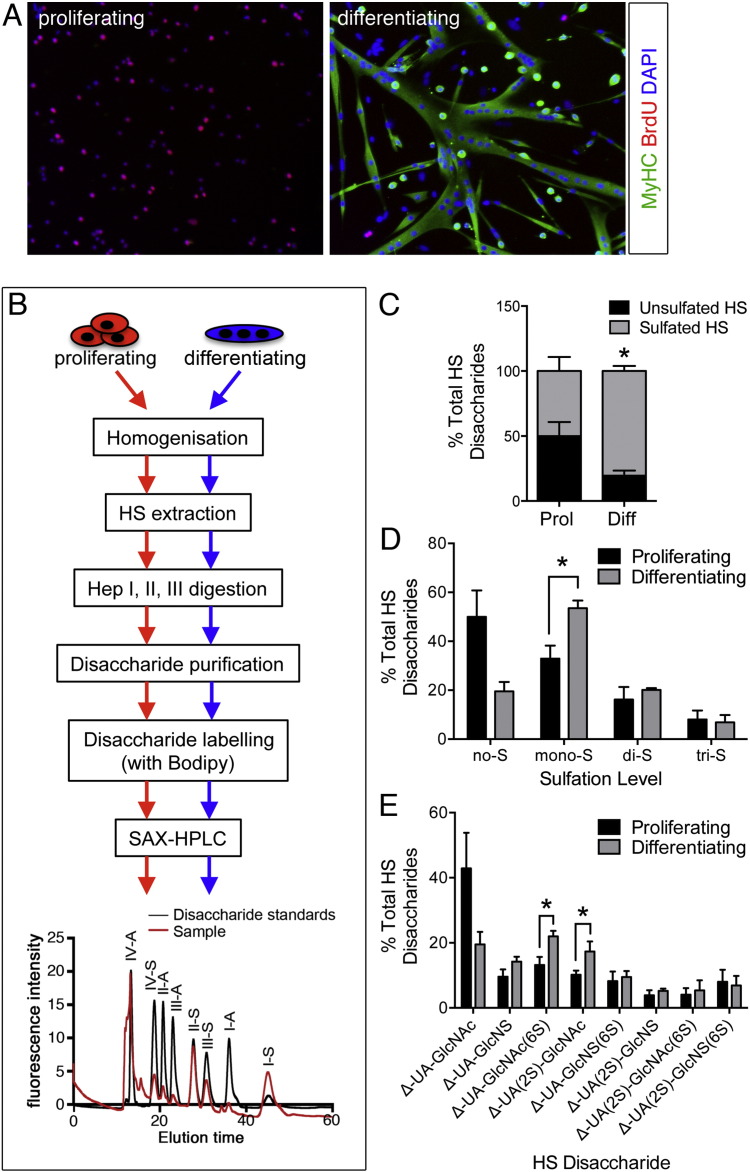
SC HS structure is dynamically regulated during myogenic differentiation. A) Representative images of proliferating and differentiating primary satellite cell-derived myoblasts where proliferation is detected via BrdU incorporation and immunostaining (red), while differentiation is identified via expression of myosin heavy chain (MyHC, green). B) Schematic showing the main steps in the process of HS extraction, digestion to release individual disaccharide units, labelling of the reducing end of the disaccharides, purification and profiling via HPLC from proliferating and differentiated cells. C–E) HS is more sulfated in differentiated myoblast cultures compared to proliferating myoblast cultures. In (C) the percentage of non-sulfated (Δ-UA-GlcNAc, unsulfated, black bars) and sulfated (sum of all sulfated, grey bars) disaccharides is plotted, based on the data from panel D. In (D) the percentage of non-sulfated Δ-UA-GlcNAc disaccharide (no-S) and the sum of the percentages of all mono-sulfated (mono-S), di-sulfated (di-S) and tri-sulfated (tri-S) disaccharides are plotted based on data from panel (E). In (E) each disaccharide entity is represented as percentage of the total amount of disaccharides identified as in (B) against a set of authentic disaccharide standards (Table 1) and plotted as averages of four independent experiments ± S.E.M. * = p < 0.05.

During myogenic differentiation primary SC-derived myoblasts undergo numerous morphological and molecular changes. Proteins typical of mature muscle cells, such as muscle myosins, are absent in proliferating cells but expressed in differentiating cells. In contrast, cell cycle markers such as 5-Bromo-2′-deoxyuridine (BrdU) incorporation are abundant in cultures of proliferating cells but nearly absent in cultures of differentiating cells (Fig. 1A). Moreover, differentiated cells fuse to one another and generate multinucleated cells called myotubes, which are the *ex vivo* equivalent of young, immature, muscle fibres (Fig. 1A). HS controls many signalling pathways involved in differentiation of muscle progenitors and thus, we hypothesised that the SC heparanome may change during differentiation, and potentially be a factor controlling the balance between proliferation and differentiation. To test this hypothesis we extracted and profiled HS from proliferating and differentiating primary SC-derived myoblasts (Fig. 1B) and quantified their abundance with reference to a set of authentic disaccharide standards (Table 1). While in cultures of proliferating myoblasts there was an even distribution between non-sulfated disaccharides and sulfated disaccharides, in differentiated cultures this balance was markedly altered, with an increase in sulfated disaccharides at the expense of non-sulfated disaccharides (Fig. 1C).

When the relative abundance of mono-sulfated, di-sulfated and tri-sulfated disaccharides was quantified, we observed a statistically significant increase in mono-sulfated disaccharides, which was accompanied by a marked decrease in non-sulfated disaccharides though the latter did not reach statistical significance (Fig. 1D).

Thus, during SC differentiation, the overall sulfation levels of HS increase, a phenomenon that is reminiscent of the increase in HS sulfation previously reported to occur during differentiation of embryonic stem (ES) cells into embryoid bodies [36] and during ES cell neural differentiation [37]. Moreover, it has been shown that genetic targeting of NDST1 and NDST2, the two enzymes that promote de-acetylation and sulfation of N-acetyl-glucosamine moieties in HS [38], inhibits ES cell adipogenic and neural differentiation [39]. Similarly, a lack of HS inhibits neural and haematopoietic differentiation of ES cells [40], [41]. In contrast, functional reduction of HS sulfation promotes de novo ES cell derivation [42]. Thus, there is increasing evidence that the sulfation level of HS in stem cell niches is a major factor contributing to stem cell fate. In this context our findings reveal that SCs are an additional stem cell type whose differentiation is associated with increased HS sulfation.

Most notably the increase in HS sulfation that we observed during primary SC-derived myoblast differentiation was specifically due to an increase in both Δ-UA-GlcNAc(6S) and Δ-UA(2S)-GlcNAc (Fig. 1E). Although the total amount of 6-O-sulfation does not appear to significantly change in differentiating versus proliferating SCs (data not shown), the observed increase in one of the 6-O-sulfated disaccharides is interesting since we had previously shown that the extracellular sulfatases, Sulf1 and Sulf2, which remove 6-O-sulfation from HS, are dramatically downregulated during SC activation and differentiation *in vivo* [22]. This downregulation of Sulfs during SC activation is consistent with our finding here that the levels of Δ-UA-GlcNAc(6S) increase during SC differentiation *in vitro* (Fig. 1E) although it apparently contrasts with findings by Langsdorf et al. that mice lacking both Sulf1 and Sulf2 show delayed SC differentiation *in vivo* upon muscle injury [43]. However, these data may not be discordant since compensation mechanisms are likely acting in the more extreme scenario of *Sulf1/2*^*−/−*^ mice and this should be taken into account. Indeed, in *Sulf1/2*^*−/−*^ SC cultures two of the three 6-O-sulfated disaccharides analysed were decreased and only one was increased [43], further supporting the widespread idea that it is not possible to directly predict changes in HS structure based simply on changes in the expression levels of the biosynthetic enzymes.

### The muscle heparanome is altered with ageing

**Fig. 2:**
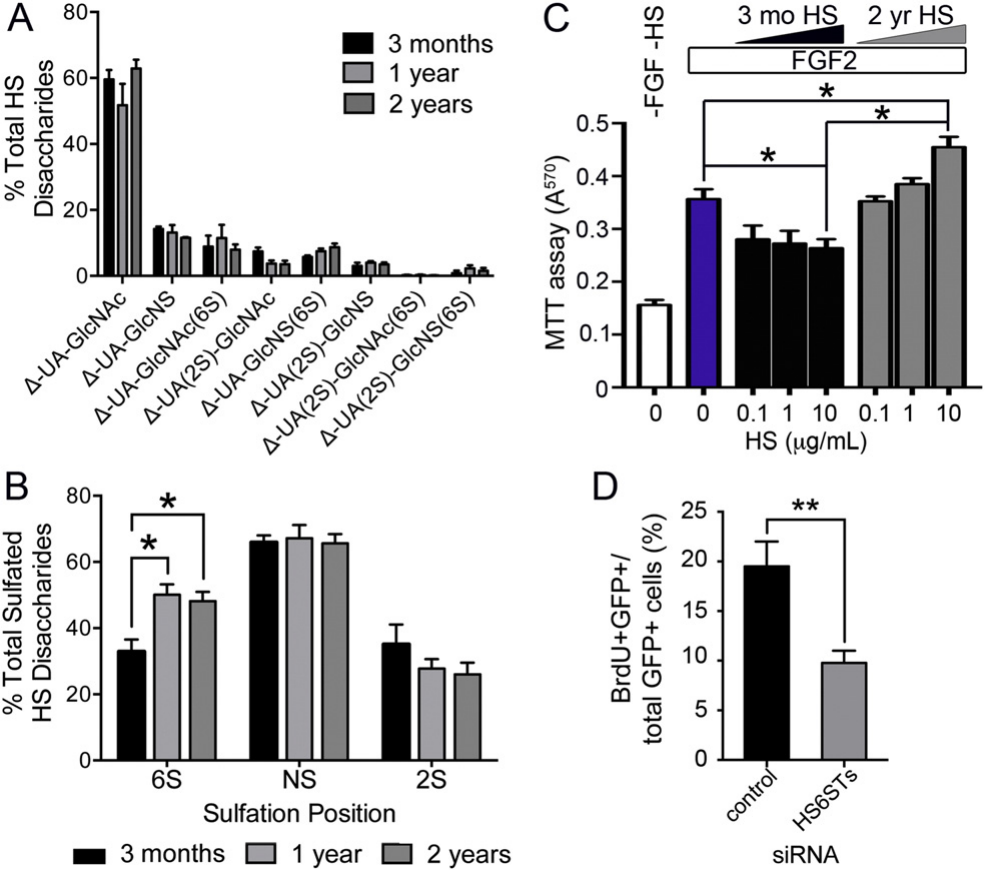
Muscle HS shows an age-associated increase in 6-O-sulfation which promotes FGF2 signalling and myoblast proliferation. Quadriceps muscles from 3 month-old (black bars), 1 year-old (light grey bars) and 2 year-old (dark grey bars) were treated and analysed as in Fig. 1A to profile HS composition. A) No significant differences in the relative abundance of individual disaccharide units were observed. B) Analysis of the abundance of specific sulfate groups: 6-O-sulfation (6S), N-sulfation (NS) and 2-O-sulfation (2S) reveals a significant increase is 6-O-sulfation in the muscle of 1 year-old and 2 year-old mice compared to the muscle of 3 month-old mice. Muscles from at least 4 animals (n = 5 in the 2 years age group, n = 4 in the 3 months and 1 year age groups) were analysed and plotted as averages ± S.E.M. C) BaF3 cell assay where the mitogenic activity of FGF2 on BaF3 cells was measured in response of FGF2 alone or in the presence of FGF2 and HS extracted from the muscles of either 3 month-old or 2 year-old mice. Data from 3 independent experiments with 4 technical replicates were analysed and plotted as averages ± S.E.M. D) GFP (pClover) and siRNA simultaneously to the three HS6STs or control scrambled siRNA were transfected into C2C12 myoblasts and 24 h later 10 μM BrdU was added to the culture medium 2 h prior to fixation and immunostaining to detect GFP and BrdU. Simultaneous knockdown of all three HS6STs produces a significant decrease in C2C12 myoblast proliferation. * = p < 0.05, ** = p < 0.01.

The number of quiescent SCs and their regenerative potential are reduced with ageing [31], [33] and accompanied by alterations in MAPK signalling [29], [30], which is strongly affected by the extracellular heparanome [44], [45]. Furthermore, previous studies have shown age-related changes in HS structures in other tissues [34], [35]. Thus, we reasoned that changes in the muscle heparanome might also be an important underlying factor in the signalling alterations observed in aged SCs. To investigate ageing-associated changes in the muscle heparanome, we profiled HS from 3-month-, 1-year- and 2-year-old mouse muscles. Although the relative abundance of individual disaccharides did not change significantly with age (Fig. 2A), we observed an overall increase in 6-O-sulfation in the quadriceps of older mice (1-year- and 2-year-old) compared to young mice (3-month-old), while 2-O-sulfation only showed a trend towards a decrease that did not reach statistical significance (Fig. 2B). Importantly, the changes observed in 6-O-sulfation were already detectable in 1-year old mice. Since sarcopenia and muscle fibrosis are not present at 12 months of age [46], our data indicate that important changes at the molecular level are already occurring in the muscle extracellular environment before any macroscopic changes at the tissue or functional level are detected. Moreover, we speculate that the increase in 6-O-sulfation that we observe in aged muscle could have important consequences on the level of activation of resident SCs. It has been reported that FGF2 signalling is increased in aged muscle and leads to SC hyper-activation and consequent exhaustion [31], [33]. Since 6-O-sulfation promotes FGF2 signalling [47], [48], it is reasonable to speculate that the increase in FGF2 signalling observed in aged SCs is, at least in part, due to the increased 6-O-sulfation that we observed in the muscle heparanome. To test this hypothesis we used a BaF3 cell assay which takes advantage of the absence of HS in BaF3 lymphoid cell cultures where an exogenous source of HS is required in order to promote FGF signalling [10]. We employed a BaF3 cell line stably transfected to express the FGF receptor 1, which is the main FGF receptor expressed by myoblasts [49] and HS extracted from 3 month-old and 2 year-old mice to study the role of ageing on HS-mediated regulation of FGF2 mitogenic activity as a measure of FGF2 signalling. When cells were treated with HS extracted from 3 month-old mice baseline FGF2 signalling, as measured by the mitogenic activity of FGF2 on BaF3 cells, was not enhanced at 0.1 and 1 μg/ml doses, and was partially reduced at the 10 μg/ml dose (Fig. 2C). In contrast, when cells were treated with HS extracted from 2 year-old mice, the mitogenic activity of FGF2 was increased significantly at the highest dose administered (10 μg/ml dose) compared to cells treated with FGF2 alone (Fig. 2C). These data strongly support a key role for aged muscle HS in the loss of quiescent SCs observed in mouse muscle with ageing and, together with the observation that changes in HS structure are already present in 1 year-old mice, suggest that an increase in FGF2 signalling occurs early in the ageing process and leads over time to exhaustion of SCs, and subsequent accumulation of muscle fibrosis and sarcopenia [50].

We have shown that 6-O sulfation is increased in aged muscle (Fig. 2B) and that this increase in 6-O-sulfation is associated with increased FGF2 mitogenic activity in a BaF3 cell assay (Fig. 2C). To further test the role of 6-O sulfation in myoblast proliferation we used RNAi technology to knock-down the three murine 6-O-sulfotransferases (HS6ST1, HS6ST2 and HS6ST3) in myoblasts (Supplementary Fig. S1) and study proliferation in response to reduction in 6-O-sulfation. When all three HS6STs were knocked-down simultaneously (Fig. 2D), myoblast proliferation was significantly decreased by approximately 50%, further supporting the hypothesis that 6-O sulfation promotes myoblast proliferation and that the observed age-associated increase in 6-O sulfation might be responsible for satellite cell hyper-activation and consequent exhaustion via FGF2.

### Exogenous HS mimetics differentially regulate SC fate partly via regulation of FGF2 signalling

**Fig. 3:**
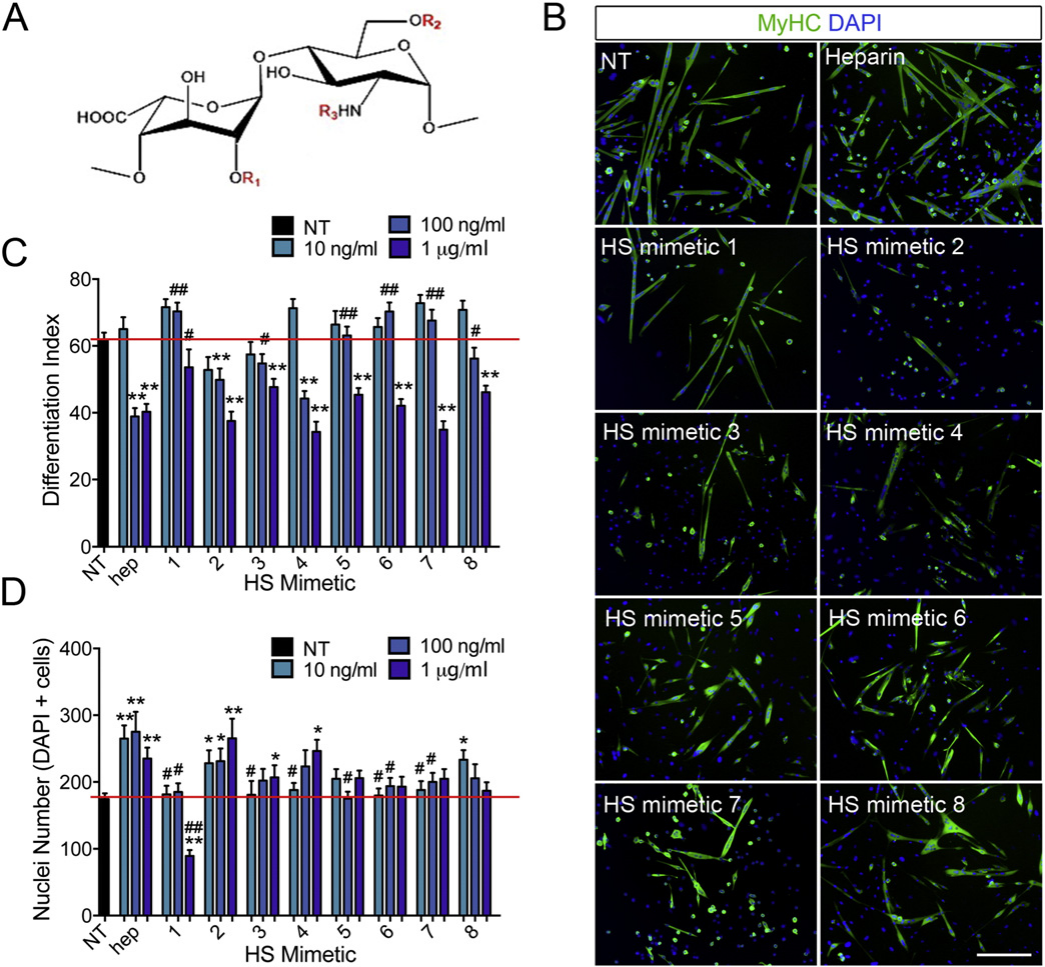
HS mimetics differentially affect myoblast differentiation and cell numbers. A) Schematic of the most abundant disaccharide unit in heparin containing iduronic acid and N-Acetyl-glucosamine (IdoA-GlcNAc) with the three main sites of sulfation indicated in red as R1 (2-O-sulfation), R2 (6-O-sulfation), R3 (N-sulfation). B) Representative images of primary SC-derived myoblasts cultured for 2 days and then induced to differentiate for an additional 3 days. Differentiated cells, both mono- and multi-nucleated are identified via immunostaining for myosin heavy chain (MyHC, green). Nuclei are identified via staining with DAPI (blue). Scale bar = 200 μm is equal across all images. C) For each condition shown in (B) the differentiation index was calculated as the percentage of nuclei in MyHC + cells (both mono- and multi-nucleated) over the total number of nuclei (DAPI +). The average of ten images across two technical replicates for three independent experiments ± S.E.M. is calculated and plotted. D) For each condition shown in (B) the total number of nuclei was measured and the average of ten images across two technical replicates for three independent experiments ± S.E.M. plotted. Asterisks are p-values for each condition compared to non-treated (NT) cells. ** = p < 0.01; * = p < 0.05. Hash signs are p-values for each condition compared to heparin-treated cells. ## = p < 0.01; # = p < 0.05.

To model in a systematic and controlled way on how changes in the composition of the SC niche heparanome might influence myogenic differentiation, we produced a library of diversely sulfated HS mimetics by selective chemical modification of heparin [51]. Heparin is a type of HS containing a high degree of sulfation in all three key positions (2S, 6S, NS), and can be exploited as a starting material to generate HS mimetics containing increased or decreased levels of sulfation in specific positions (Fig. 3A and Table 2). Through this process we obtained a library of HS mimetics containing lower levels of sulfation than native heparin, distributed in all possible combinations of the three types of HS sulfation, and therefore mimicking the variant types of HS normally found in tissues: completely de-sulfated HS (HS mimetic 8), HS with sulfation in only one position (HS mimetic 5, HS mimetic 6 and HS mimetic 7), HS with sulfation in two positions (HS mimetic 2, HS mimetic 3 and HS mimetic 4). Additionally, we produced HS with sulfation in more than the three canonical positions (oversulfated heparin, HS mimetic 1, in which additional 3-O-sulfate groups are present on glucosamine and uronic acid rings) (Table 2). We then asked how altering the SC extracellular heparanome via addition of specific exogenous HS mimetics affects myogenic differentiation.

SC-derived primary myoblasts were treated with the HS mimetics generated as above concomitant with induction of myogenic differentiation (see *Methods* section for details). We found that the effect of different HS mimetics on myoblast differentiation was structure-specific and concentration-dependent with the lowest doses tested often leading to non-significant differences compared to absence of treatment (Fig. 3B–C). However, at the higher concentrations tested (100 and 1000 ng/ml) almost all the HS mimetics, except oversulfated heparin (HS mimetic 1), produced a marked reduction in differentiation (Fig. 3B–C). This general reduction in differentiation was accompanied by an overall increase in cell numbers (Fig. 3B, D) with the HS mimetics that decreased differentiation the most (HS mimetic 2, HS mimetic 3 and HS mimetic 4) showing also the greatest effects on increasing cell numbers (Fig. 3B–D). In myoblasts, differentiation and proliferation are mutually exclusive and thus, these observations suggest that addition of HS in the presence of low serum increases the activity of pro-proliferative and/or anti-differentiative signals, which in turn delays differentiation and promotes cell expansion. The exception to this general trend was provided by oversulfated heparin (HS mimetic 1), which did not reduce differentiation even at high concentration (Fig. 3B–C), but dramatically reduced cell numbers (Fig. 3B, D). It was unlikely that this reduction in cell numbers was due to cell detachment or cell death, since no detached or dead cells were observed. Instead it was likely due to induction of cell cycle arrest. The interesting finding that oversulfated heparin (HS mimetic 1) produces cell cycle arrest is consistent with the observation that sulfation levels of endogenous HS increase when SC-derived myoblasts exit the cell cycle to differentiate (Fig. 1).

We then studied whether the HS mimetics affected myoblast fusion by scoring the percentage of differentiated (expressing myosin heavy chain, MyHC) cells found in multinucleated myotubes. Not surprisingly myoblast fusion was affected by the presence of HS mimetics in a similar way to myoblast differentiation (Supplementary Fig. S2), suggesting that the heparanome is not directly involved in the signalling pathways that control myoblast fusion.

We have shown that muscle ageing is associated with an increase in 6-O-sulfation (Fig. 2) and it is well established that aged SCs are impaired [29], [30], [32], [52], [53]. Thus, it is intriguing to ask whether any of the HS mimetics tested could be considered a proxy for aged muscle HS. An increase in 6-O sulfation might be mimicked by 2-de-sulfated-N-acetylated heparin (HS mimetic 5), which contains a greater proportion of 6-O-sulfation as a result of selective de-sulfation of the 2-O and N positions. This HS mimetic indeed inhibits myogenesis by inhibiting myoblast differentiation without promoting proliferation (Fig. 2B–D) further supporting a key role for HS-mediated signalling in age-associated SC impairment.

### FGF2 signalling via Erk1/2 and mitogenic activity are differentially affected by different HS structures in myoblasts

**Fig. 4:**
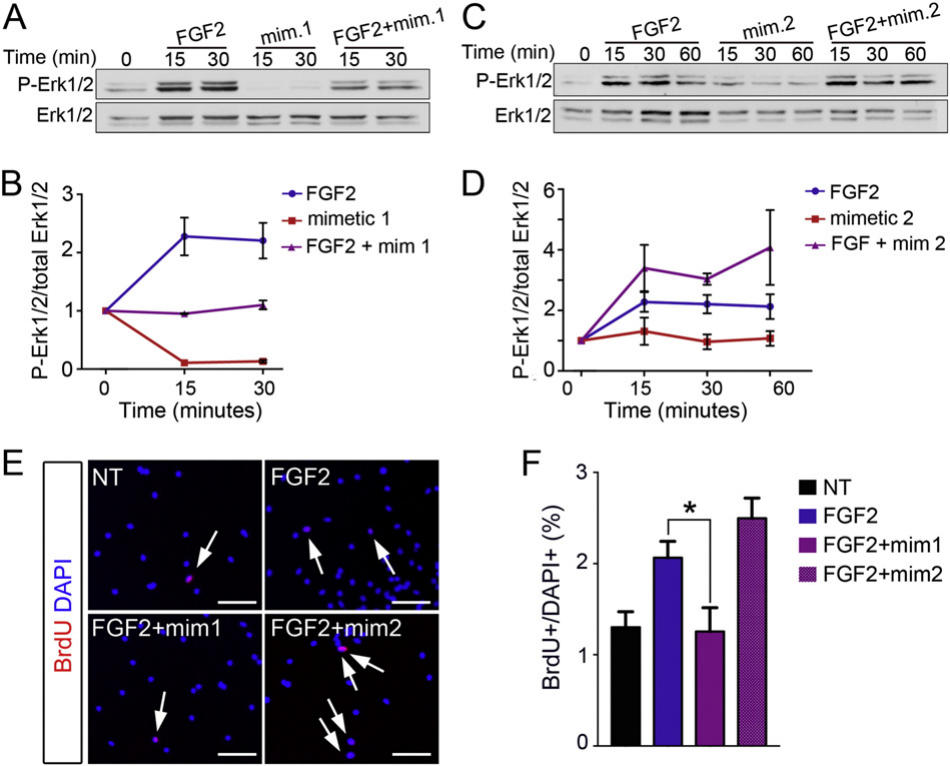
FGF2 signalling via Erk1/2 and mitogenic activity are differentially regulated by HS mimetics. A) C2C12 myoblasts were serum-starved for 6 h (lane 1) then stimulated with either 2 nM FGF2 alone (lanes 2, 3) or HS mimetic 1 alone (lanes 4, 5) or FGF2 + HS mimetic 1 (1 μg/ml) together (lanes 6, 7) for the indicated amounts of time. At the end of the stimulus cells were immediately lysed and analysed via Western blotting to detect phosphorylated Erk1/2 (P-Erk1/2) and total Erk1/2. One representative of three independent experiments is shown. B) Quantification of phospho-Erk1/2 band intensity normalised to total Erk1/2 intensity. Moreover, all time points were then normalised to time zero (end of serum starvation) and the averages of three independent experiments plotted ± S.E.M., except for the condition “FGF2 alone at 15 and 30 minutes” which were averaged across 6 independent experiments. C) C2C12 myoblasts were serum-starved for 6 h (lane 1) then stimulated with either 2 nM FGF2 alone (lanes 2, 3, 4) or HS mimetic 2 (1 μg/ml) alone (lanes 5, 6, 7) or FGF2 + HS mimetic 2 (lanes 8, 9, 10) for the indicated amounts of time. At the end of the stimulus cells were immediately lysed and analysed via Western blotting to detect phosphorylated Erk1/2 (P-Erk1/2) and total Erk1/2. One representative of three independent experiments is shown. D) Quantification of phospho-Erk1/2 band intensity normalised to total Erk1/2 band intensity, moreover all time points were then normalised to time zero (end of serum starvation) and the averages of three independent experiments plotted ± S.E.M., except for the condition “FGF2 alone at 15 and 30 min” which were averaged across 6 independent experiments. E) Primary SC-derived myoblasts were expanded for two days in growth medium then serum-starved overnight to induce cell cycle exit followed by treatment with either vehicle (NT), FGF2, FGF2 + HS mimetic 1 or FGF2 + HS mimetic 2. F) Quantification of (E) where ten random fields across three biological replicates were scored. Scale bars are = 100 μm, * = p < 0.05.

FGF2 strongly promotes myoblast expansion by inhibiting differentiation and requires HS to signal [16], [54], [55], [56]. Moreover, FGF2 signalling is sensitive to the degree and pattern of HS sulfation [10]. Since we have shown that: (i) myoblast differentiation is associated with increased sulfation levels (Fig. 1) and (ii) oversulfated heparin (HS mimetic 1) is the only HS mimetic that does not inhibit differentiation but inhibits cell expansion, we hypothesised that oversulfated heparin might inhibit FGF2 signalling in myoblasts by increasing the total amount of sulfated HS in the extracellular environment. To test this hypothesis, we treated myoblasts with FGF2 or oversulfated heparin (HS mimetic 1) or a combination of the two after a period of serum starvation and quantified the cellular response by measuring Erk1/2 phosphorylation levels by Western blotting. As expected, FGF2 addition produces an increase in the levels of phospho-Erk1/2 as measured 15 and 30 min after stimulation (Fig. 4A–B). In contrast, the addition of HS mimetic 1 together with FGF2 does not produce an increase in Erk1/2 phosphorylation, supporting the hypothesis that HS mimetic 1 blocks FGF2 signalling (Fig. 4A–B). Moreover, the addition of HS mimetic 1 alone produces a dramatic decrease in phospho-Erk1/2 after 15 and 30 min compared to the zero time point, suggesting that the oversulfated HS mimetic 1 inhibits Erk1/2 signalling also in response to endogenous paracrine signals (Fig. 4A–B).

In contrast to oversulfated heparin, the HS mimetic N-acetylated heparin (HS mimetic 2) produced the strongest opposing effect on myoblasts: it increased cell numbers and dramatically reduced differentiation (Fig. 3B–D). To test whether the anti-differentiative and/or pro-proliferative effect of HS mimetic 2 on differentiating myoblasts was also mediated by FGF2, we treated myoblasts with FGF2 in the presence or absence of HS mimetic 2, after a period of serum starvation and quantified the levels of Erk1/2 phosphorylation. We observed that myoblast response to FGF2 was only modestly enhanced by the presence of HS mimetic 2, but, importantly, it was prolonged over time: at 60 min of FGF2 stimulation the levels of phospho-Erk1/2 were higher in the presence of HS mimetic 2 compared to its absence (Fig. 4C–D). This observation that HS mimetic 2 potentiates FGF2 signalling further supports the hypothesis that the mechanism of action of HS mimetics on primary myoblasts involves, at least in part, regulation of FGF2 signalling via Erk1/2.

To further explore the role of specific HS structures on FGF2 signalling in myoblasts, we serum-starved proliferating primary SC-derived myoblasts overnight to induce cell cycle exit and then added FGF2, either alone or in combination with HS mimetics 1 and 2, to study their role in regulating FGF2-induced re-entry into the cell cycle. As expected, the number of cells re-entering the cell cycle upon FGF2 stimulation was decreased in the presence of HS mimetic 1 (Fig. 4E–F), which we had previously shown to reduce FGF2 signalling (Fig. 4A–B), and increased in the presence of HS mimetic 2 (Fig. 4E–F), which we had shown increases FGF2 signalling (Fig. 4C–D), compared to cells treated with FGF2 alone (Fig. 4E–F). Taken together these data strongly support a role for specific HS structures in regulating myoblast proliferation via regulation of FGF2 signalling.

### Different HS structures differentially regulate SC self-renewal

**Fig. 5:**
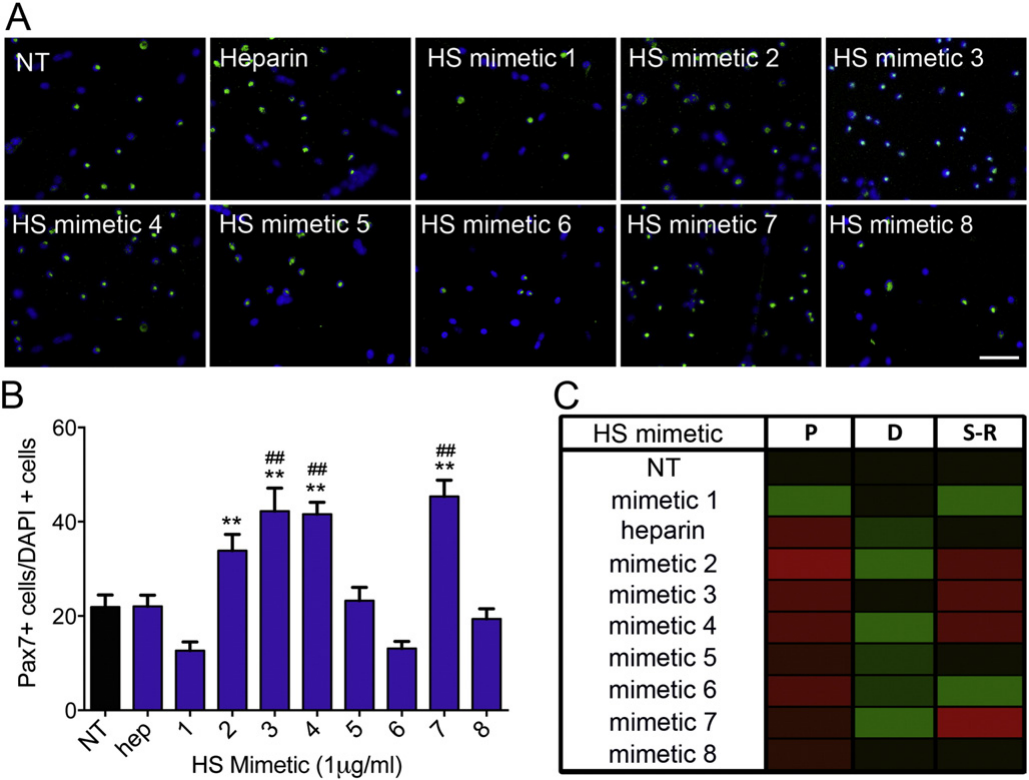
Varied HS structures differentially affect satellite cell self-renewal *ex vivo*. A) Representative images of SC-derived myoblasts cultured for 2 days and then induced to differentiate for an additional 3 days. Reserve cells are identified via immunostaining for Pax7 (green). Nuclei are identified via staining with DAPI (blue). B) For each condition shown in (A) the percentage of Pax7 + nuclei over the total number of nuclei was calculated and plotted as the average of ten images across two technical replicates, each for three independent experiments ± S.E.M. ** = p < 0.01. C) Schematic summary of the effect produced by HS mimetics on primary SC-derived myoblasts induced to differentiate. Keys: P = proliferation, D = differentiation, S–R = self-renewal.

Lastly, we tested whether different HS mimetics affected SC self-renewal. In the muscle tissue, the steady state number of undifferentiated progenitors is determined by the equilibrium between the number of cells that become activated and proceed through the myogenic lineage and the number of cells that return quiescent upon activation, a process called self-renewal [12]. In primary SC-derived myoblast cell culture models the process of SC self-renewal is recapitulated when, during differentiation, a population of cells exits the cell cycle but instead of differentiating enters a quiescent state similar to that of quiescent SCs in the intact muscle. These self-renewing cells in culture are called reserve cells and express the transcription factor Paired Box 7 (Pax7) [57]. To test whether changes in the extracellular heparanome affected SC self-renewal, we quantified the numbers of Pax7 + cells in SC-derived myoblast cultures that had been induced to differentiate and treated with the highest concentration (1000 ng/ml) of HS mimetics previously tested for differentiation and cell expansion (Fig. 5A–B). Although at this concentration all the HS mimetics tested, except oversulfated heparin, produced a decrease in SC differentiation, the effect on SC self-renewal was variable. For example, HS mimetic 6 and HS mimetic 7 produced comparable effects on differentiation and cell expansion but completely opposite effects on SC self-renewal, with HS mimetic 6 inhibiting self-renewal and HS mimetic 7 promoting self-renewal (Fig. 5C). These observations strongly support the view that HS regulates satellite cell fate in a complex manner that cannot be simplified to a dichotomy between HS structures that promote or inhibit differentiation. This is typical of HS functions, since HS is a highly complex biomolecule involved in the simultaneous regulation of several signalling pathways.

Overall, in this study we show that a correlation between structure and function of HS in myogenesis exists as demonstrated by: (i) significant changes in endogenous HS during differentiation and ageing; (ii) matching differential effects of different extracted HS or semi-synthetic HS mimetics on satellite cell fate decisions and on regulating signalling pathways relevant to SC fate; this was exemplified by FGF2 signalling, though effects on other pathways cannot be excluded by our data. Although more investigation is needed to further clarify the role of the heparanome in myogenesis, in health, ageing and disease, our data clearly indicate the heparanome as an important dynamic and regulatory component for SC regulation.

## Experimental procedures

### Mice

Young (11–13 weeks) and old (1 year and 2 years) C57Bl/6J female mice were obtained from Charles River UK, housed in a pathogen-free facility at the University of Liverpool, UK and used in accordance with the Animals (Scientific Procedures) Act 1986 and the EU Directive 2010/63/EU and after local ethical review and approval by Liverpool University's Animal Welfare and Ethical Review Body (AWERB).

### Cell culture

SC-derived primary myoblasts were obtained by expansion in culture of primary SCs, which were obtained, expanded and differentiated as described previously [20] from 11 to 13 week old female mice (*Charles River*) and plated either on gelatin-coated 10 cm plates (for HSPG extraction, see details below) or on gelatin-coated 12 multi-well plates (for screening of HS mimetics, see details below). SC-derived myoblasts were expanded in growth medium (F12 + 0.4 mM CaCl_2_ + 15% horse serum (*HyClone*) + 1% penicillin/streptomycin + 2 mM glutamine + 2 nM FGF2) under a humidified atmosphere of 5% CO_2_. Recombinant human FGF2 was prepared in house. To induce differentiation, cell cultures were washed twice with F12C (F12 + 0.4 mM CaCl_2_) then incubated in differentiation medium (F12 + 0.4 mM CaCl_2_ + 3% horse serum (*HyClone*) + 1% penicillin/streptomycin + 2 mM glutamine) for three days.

For BrdU incorporation analysis, 10 μM BrdU was added to the culture medium 2 h prior to cell fixation to label all cells that were in S-phase.

For biochemistry experiments primary SCs were replaced with C2C12 myoblasts to increase the amount of starting material. C2C12 myoblasts [58], [59] were obtained from ATCC, grown in growth medium (Dulbecco's Modified Eagle Medium (*Invitrogen*) + 10% foetal bovine serum (*Invitrogen*) + 1% penicillin/streptomycin + 2 mM glutamine) under a humidified atmosphere of 5% CO_2_. Cells were maintained at 40–70% confluence on uncoated plastic dishes. For Western blot analysis, cells were serum starved for 6 h, treated with the HS mimetic and/or FGF2 and then, at the indicated time points, lysed and processed for Western blotting as described below.

### Differentiation and treatment with modified heparins

Chemically modified heparin compounds (HS mimetics) were prepared and kindly donated by Dr. Ed Yates, University of Liverpool. Primary SCs were induced to differentiate by switching to differentiation medium (DMEM + 3% horse serum + 1% penicillin/streptomycin + 2 mM glutamine) in the presence/absence of HS mimetics for 3 days prior to fixation with 4% paraformaldehyde prepared in phosphate buffered saline (PBS) for 10 min at room temperature and then washed 3 times with PBS. Fixed cells were stored in PBS at 4 °C until immunostaining was performed.

### Immunofluoresence

For detection of myosin heavy chain (MyHC) and Pax7, fixed cells were permeabilized with PBS + 0.2% TritonX100 for 10 min at room temperature. For BrdU immunostaining, after permeabilization the DNA was denatured with 3 N HCl for 20 min at RT followed by a 10 min incubation with 0.1 M sodium borate, pH 8.5 and then three washes in PBS. For all immunostaining procedures, samples were then blocked with 3% bovine serum albumin (BSA) for 1 h before incubation with primary antibodies over night at 4 °C in PBS + 1% BSA. Primary antibodies used were: mouse monoclonal anti-MyHC (*Developmental Studies Hybridoma Bank at Iowa University*, MF20 clone) at 1:200, mouse monoclonal anti-Pax7 (*Developmental Studies Hybridoma Bank at Iowa University*) at 1:100 and rat anti-BrdU (Serotec) at 1:100. Cells were then washed 3 times with PBS + 0.2% TritonX100 and incubated for 1 h at room temperature with PBS + 1% BSA + secondary antibodies conjugated with AlexaFluor 488 (*Invitrogen*) at 1:500 followed by incubation with 2 μg/ml DAPI (*Life Technologies*) in PBS for 5 min at room temperature. Cells were then washed 3 times with PBS + 0.2% TritonX100 then stored at 4 °C in PBS until imaging on an epifluorescence microscope (EVOS-FL, *Life Technologies*) using a 10 × objective.

### Extraction of HSPGs and disaccharide analysis

Quadriceps muscles were homogenized with a blade homogenizer and solubilized in 1 ml of TUT buffer (1% Triton × 100, 8 M urea, 10 mM Trizma Base, 0.1 mM Na_2_SO_4_, pH 8.0), then cleared by centrifugation at 13,000 ×* g* for 10 min. For HSPG extraction and disaccharide analysis from primary myoblasts, cells were washed twice with ice-cold PBS, solubilized in 1 ml TUT buffer/10 cm^2^ culture dish and sonicated to disrupt DNA. Cell or muscle homogenates were added to diethylaminoethyl (DEAE) Sephacel beads (GE healthcare) and incubated over night at 4 °C on gentle mixing. The DEAE beads were washed with 10 volumes of PBS, pH 7.4 followed by 10 volumes of PBS + 0.3 M NaCl, pH 7.4 and then eluted with 10 volumes of PBS + 2 M NaCl, pH 7.4. Eluted fractions were desalted on HiTrap Desalting Columns (*GE healthcare)* or with SnakeSkin Dialysis Tubing (34 mm dry flat width, *ThermoScientific*) and freeze-dried prior to resuspension in 100 mM sodium acetate, 0.1 mM calcium acetate, pH 7.0 and and digestion by sequential addition at 37 °C of 2.5 mU of heparinase I (2 h, 37 °C) followed by 2.5 mU of heparinase III (2 h, 37 °C) and finally with 2.5 mU of heparinase II (2 h, 37 °C). All three enzymes were then added together at 2.5 mU each (16 h, 37 °C). The enzymes were denatured at 95 °C for 5 min and ionic interactions were disrupted with 2 M NaCl. The enzymes and core proteins were removed using C18 Spin Columns (*Life Technologies*) and the salt removed using Graphite Spin Columns (*Life Technologies*). The samples were then freeze-dried and then resuspended in BODIPY hydrazide (5 mg/ml in methanol). Methanol was removed by centrifugation under vacuum, and samples were resuspended in DMSO: acetic acid (17:3 v/v) prior to incubation for 4 h at room temperature in the dark. Samples were snap frozen and freeze dried. Excess free BODIPY tag was removed by thin layer chromatography (TLC), bound labelled disaccharides were eluted from silica scrapings with deionized water (3 × 4 ml) and then freeze-dried. 800 μl of saturated ethanol solution (sodium acetate in molecular biology grade ethanol) was added to lyophilised samples, incubated on ice for 15 min and then centrifuged at 13,000 ×* g* for 5 min. The supernatant was collected and dried by centrifugation under vacuum. The fluorescently labelled disaccharides were resuspended in H_2_O prior to loading onto a Propac PA-1 column and eluted using a linear gradient of 0–1 M sodium chloride (prepared in 150 mM NaOH) over 30 min at a flow rate of 1 ml/min on a Shimadzu HPLC system. Peaks were detected using inline fluorescence detection at excitation wavelength of 488 nm and an emission wavelength of 520 nm using a Shimadzu RF-551 detector. The column was reconditioned by washing with 2 M NaCl (in 300 mM NaOH) before equilibrating in 150 mM NaOH. Disaccharides were identified with reference to authentic heparin unsaturated disaccharide standards (*Dextra Labs*, Table 1). Previously calculated correction factors were applied to quantitate the observed disaccharides.

### BaF3 cell assay

Murine BaF3 lymphocytes expressing murine FGFR1IIIc were a gift from Professor David Ornitz (Washington University, St. Louis, USA). Cells were maintained in RPMI-1640 supplemented + 10% FCS + 100 Units/ml penicillin G + 100 μg/ml streptomycin sulfate (all from ThermoFisher, UK) and 1 ng/ml murine interleukin-3 (R&D Systems, UK) at 10^5^ to 10^6^ cells/ml at 37 °C, 5% CO_2_. For assays, cells were plated at 10^5^ cells/ml in 96-well, flat bottomed Costar tissue culture plates (Corning, USA) in 100 μl growth medium without interleukin-3 supplemented with FGF-2 (R&D Systems, UK) and heparan sulfate at the indicated concentrations. Cells were incubated for 72 h at 37 °C, 5% CO_2_. Thiazolyl blue tetrazolium bromide (Sigma, UK) was then added to a final concentration of 250 μg/ml and the cells were incubated a further 4 h at 37 °C, 5% CO_2_. The assay was stopped with the addition of 50 μl 10% SDS, 0.01 N HCl and the plates were incubated 4 h to overnight at 37 °C to dissolve the formazan product. Plates were read at 570 nm using a Thermo multiskan EX plate reader (ThermoFisher, UK).

### Purification of HS

Muscle homogenates were added to diethylaminoethyl (DEAE) Sephacel beads (GE Healthcare) and incubated over-night at 4 °C on gentle mixing. The DEAE beads were washed with 10 volumes of PBS, pH 7.4 followed by 10 volumes of PBS + 0.3 M NaCl, pH 7.4 and then eluted with 10 volumes of PBS + 2 M NaCl, pH 7.4. Eluted fractions were desalted on HiTrap Desalting Columns (GE Healthcare) or with SnakeSkin Dialysis Tubing (34 mm dry flat width, ThermoScientific) and freeze-dried.

### Isolation of HS for activity assays

Freeze-dried samples were re-suspended in 1:5 volume of 10 × DNase solution (200 mM Tris–acetate pH 8.0, 30 mM MgCl_2,_ 50 mM CaCl_2_ with 1 μg/ml DNase was added for 4 h, 37 °C. 1:5 volume of 5 × RNase solution was then added (50 mM Tris pH 8.0, 25 mMEDTA, 40 mM Na-Acetate) with 0.5 mg/ml RNase was added for 1 h, 37 °C. 1:5 volume of 5 × Chondroitin ABC lyase solution (500 mM Tris–acetate pH 8.0) with 1.25 U cABCase was added for 4 h 37 °C. 1:5 volume of 5 × Neuraminidase buffer (250 mM Tris Acetate pH 7.5) with 2.5 mU of neuraminidase was added for 4 h, 37 °C. 1:5 volume of 5 × Keratanase buffer (250 mM Tris Acetate, pH 7.5) with 100 mU keratanse was added for 4 h, 37 °C. 1:5 volume of Pronase solution (500 mM Tris–acetate, 50 mM Calcium acetate pH 8.0) with 10 mg/ml Pronase was added for 16 h, 37 °C. The samples were then added to (DEAE) Sephacel beads to remove the enzymes. The isolated HS chains were eluted from the beads as described previously. Samples were freeze-dried and re-suspended in 100 μl filtered miliQ water. 5 μl were taken through for heparinise digestion (described above) for HS quantification. After the enzymes were removed the freeze-dried samples were re-suspended in 20 μl of miliQ water for quantification on the nano-drop measuring the absorbance of the double bond at 232 nm.

### Western blotting

Whole cell extracts were obtained by incubating cells in modified RIPA buffer (50 mM Tris–HCl, pH 7.4, 1% NP-40, 0.25% sodium-deoxycholate, 150 mM NaCl, and 1 mM EDTA) supplemented with a protease inhibitor cocktail (*Complete, Roche*) and phosphatase inhibitors (2 mM Na_3_VO_4_ and 2 mM NaF) for 30 min on ice, then cleared by centrifugation at 13,000 ×* g* for 10 min at 4 °C. Total protein content (10–30 μg) was separated on 10% SDS-PAGE gels and transferred onto nitrocellulose membranes (*Hybond, GE Healthcare*) for Western blotting. Primary antibodies were incubated overnight at 4 °C in PBS + 5% BSA and were: rabbit anti-p44/42 MAPK (Erk1/2, *Cell Signaling Technology*) used at 1:1000, rabbit anti-Phospho-p44/42 MAPK (P-Erk1/2, Thr202/Tyr204, *Cell Signaling Technology*) used at 1:1000. Anti–rabbit HRP-conjugated secondary antibody (*Santa Cruz Biotechnology*) was used at 1:10,000 in PBS + 5% BSA for 1 h at room temperature, and HRP activity was visualized using the ECL Plus system (*BioRad*). Images were quantified using the “Analyze Gel” function of *ImageJ*.

### Knockdown of HS6STs

C2C12 myoblasts were seeded in a 12-well plate at a density of 20,000 cells per well. Cells were cultured in growth medium for 24 h after which the medium was replaced with Optimem (Gibco). GFP plasmid (pClover) was co transfected 20 nM of each HS6ST siRNA using with Lipofectamine2000. siRNA sequences were obtained from Sigma Aldrich (HS6ST1, SASI_Mm01_00066864; HS6ST2, SASI_Mm02_00292422; HS6ST1, SASI_Mm01_00105592; universal negative control 1). The extent of gene knockdown was assessed by quantitative real time PCR (qPCR) using RNA purified from siRNA-treated cells 24 h post transfection. To assess proliferation of siRNA-treated cells, 22 h post transfection a BrdU proliferation assay was performed on myoblasts in the 12-well plates.

### qPCR

RNA was isolated using a Qiagen RNeasy Mini Kit (Qiagen) following manufacturer's instructions. RNA quality and integrity were checked using a Nanodrop 1000 (Thermo Fisher Scientific). Following RNA extraction, cDNA was synthesised from a maximum of 5 μg of RNA using the Tetro cDNA Synthesis Kit (Bioline). Gene expression was quantified by qPCR using the LightCycler 460 SYBR Green I Master kit (Roche) with 5 μl cDNA corresponding to 20 ng total RNA in a 20 μl final volume and 0.4 μM of each primer. Experiments were performed in duplicate for each sample in a 96-well plate (Roche). The PCR programme was set as: 95 °C for 5 min followed by 45 cycles at 95 °C for 12 s, 57 °C for 10 s, and 72 °C for 10 s. Amplification specificity was checked using a melting curve following the manufacturer's instructions. Primers were designed using the NCBI Primer-BLAST software to have a melting temperature of 60 °C and an amplicon size between 100 and 200 base pairs (Table 3). Primers were purchased from Eurofins Genomics. Results were analysed with the LightCycler software (Roche) using the second derivative maximum method to set the threshold cycle (C_T_). The relative quantitative analysis was carried out using the comparative C_T_ method (2^− ΔΔCT^) method such that expression levels of all three HS6STs were first normalised to the reference gene glyceraldehyde 3-phosphate dehydrogenase (GAPDH) and then the normalised HS6ST/GAPDH values obtained from cells transfected with HS6ST siRNAs were expressed as percentage of the HS6ST/GAPDH values obtained from cells transfected with control (scrambled) siRNA.

### Image processing, analysis and figure preparation

Photoshop 7.0 (*Adobe Systems*) was used to linearly reduce the background throughout the whole image using contrast and brightness adjustments and to compose the figures. For immunofluorescence image quantification a bespoke macro written for *Fiji* (available at [http://fiji.sc]) was applied and for each image the following data were generated: numbers of nuclei, numbers of cells expressing MyHC, numbers of nuclei present in myotubes and total myotube area. Assistance in analysis was provided by the Liverpool Centre for Cell Imaging, University of Liverpool.

### Statistical analysis

Statistical analysis was performed using GraphPad Prism (version 5.00 for Mac, GraphPad Software, San Diego California USA) and Excel (Microsoft, USA). Values are reported as the mean ± standard error of the mean (S.E.M.) for normal data. A one-way ANOVA or an un-paired t-test was used for statistical analysis. Data was deemed statistically significant when p < 0.05.

## Figures and Tables

**Table 1:** Disaccharide reference standards for SAX-HPLC characterisation of extracted HS.

Standard ID	Standard structure	Elution time
I-S	Δ-UA-GlcNAc	12.78
II-S	Δ-UA-GlcNS	21.26
III-S	Δ-UA-GlcNAc(6S)	18.59
IV-S	Δ-UA(2S)-GlcNAc	37.51
I-A	Δ-UA-GlcNS(6S)	31.14
II-A	Δ-UA(2S)-GlcNS	27.66
III-A	Δ-UA(2S)-GlcNAc(6S)	46.08
IV-A	Δ-UA(2S)-GlcNS(6S)	31.14

**Table 2:** List of HS mimetics obtained by selective chemical modification of heparin.

Modified heparin HS mimetic	Figure annotation	R1	R2	R3	Additional modification
Heparin	Heparin	SO_3_^−^	SO_3_^−^	SO_3_^−^	
Oversulfated heparin	HS mim 1	SO_3_^−^	SO_3_^−^	SO_3_^−^	SO_3_^−^ at C3 of GlcN and C3 of IdoUA
N-acetylated heparin	HS mim 2	SO_3_^−^	SO_3_^−^	COCH_3_	
2 de-sulfated-N-sulfated heparin	HS mim 3	H	SO_3_^−^	SO_3_^−^	
6 de-sulfated-N-sulfated heparin	HS mim 4	SO_3_^−^	H	SO_3_^−^	
2 de-sulfated-N-acetylated heparin	HS mim 5	H	SO_3_^−^	COCH_3_	
6 de-sulfated-N-acetylated heparin	HS mim 6	SO_3_^−^	H	COCH_3_	
2,6 de-sulfated-N-sulfated heparin	HS mim 7	H	H	SO_3_^−^	
2,6 de-sulfated-N-acetylated heparin	HS mim 8	H	H	COCH_3_	

**Table 3:** primers used for HS6ST and GAPDH qPCR and amplicon size.

Gene	Forward primer	Reverse primer	Size
GAPDH	CCCCTTCATTGACCTCAACTAC	TCCACGACATACTCAGCACC	182
HS6ST1	CGTTCGCCCAGAAAGTTCTAC	CACACATGTGCAAGGAGGTC	118
HS6ST2	CCCTTGGCCAGCGTCG	GCACACGTATTGGACGA	102
HS6ST2	GGCAAGAAGGAGACCTGGCT	GTAGAAATTCCTGGTGTGGCTGTG	150

## Appendix A Supplementary data

Supplementary figures Image 1
