# Supplementary material for: Dynamic changes in heparan sulfate during muscle differentiation and ageing regulate myoblast cell fate and FGF2 signalling
Source: Matrix Biol. 2017 May;59:54–68. doi: 10.1016/j.matbio.2016.07.007 (PMC5380652; doi:10.1016/j.matbio.2016.07.007)
Supplement: Supplementary file 1 — Supplementary figures [file mmc1.docx]

**Dynamic Changes in Heparan Sulfate During Muscle Differentiation And Ageing are Associated with Regulation of Myoblast Cell Fate and FGF2 Signalling**

**SUPPLELMENTARY FIGURES**

Ghadiali R. S., Guimond S. E., Turnbull J. E., Pisconti A*.

*Department of Biochemistry, Centre for Glycobiology, Institute of Integrative Biology, University of Liverpool, Crown Street, Liverpool L69 7ZB*

*Correspondence to:

Addolorata Pisconti, [pisconti@liverpool.ac.uk](mailto:pisconti@liverpool.ac.uk)

**
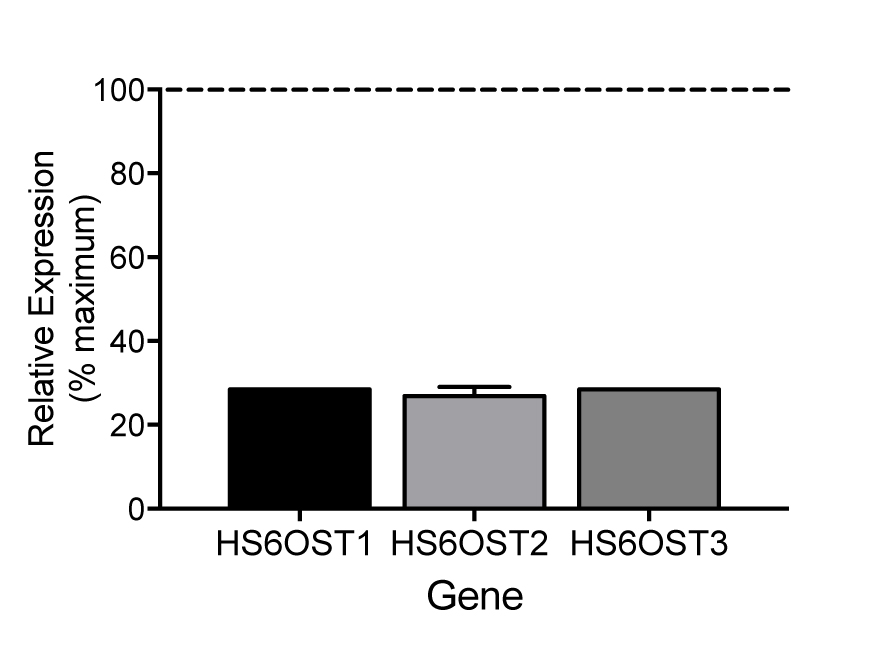
**

**Figure S1: Transfection of siRNA against HS6ST1, HS6ST2 and HS6ST3 results in knock-down of HS6ST expression by approximately 75%.** C2C12 cells were transfected with either 60 nM universal control siRNA or with a combined treatment of 20 nM of siRNA directed to each one of the three HS6STs (HS6ST1, HS6ST2 and HS6ST3). GAPDH was used as a house-keeping gene and the mRNA levels of the three HS6STs and of GAPDH measured by qPCR in both transfection conditions. The ΔΔCt method was used to quantify and normalise the results which are expressed as percentage of each HS6ST/GAPDH mRNA in the 3xHS6ST siRNA-transfected cells over the amount of each HS6ST/GAPDH mRNA in the cells transfected with control siRNA. One representative of two independent experiments is shown where the results from two technical replicates are averaged and plotted ± standard deviation.


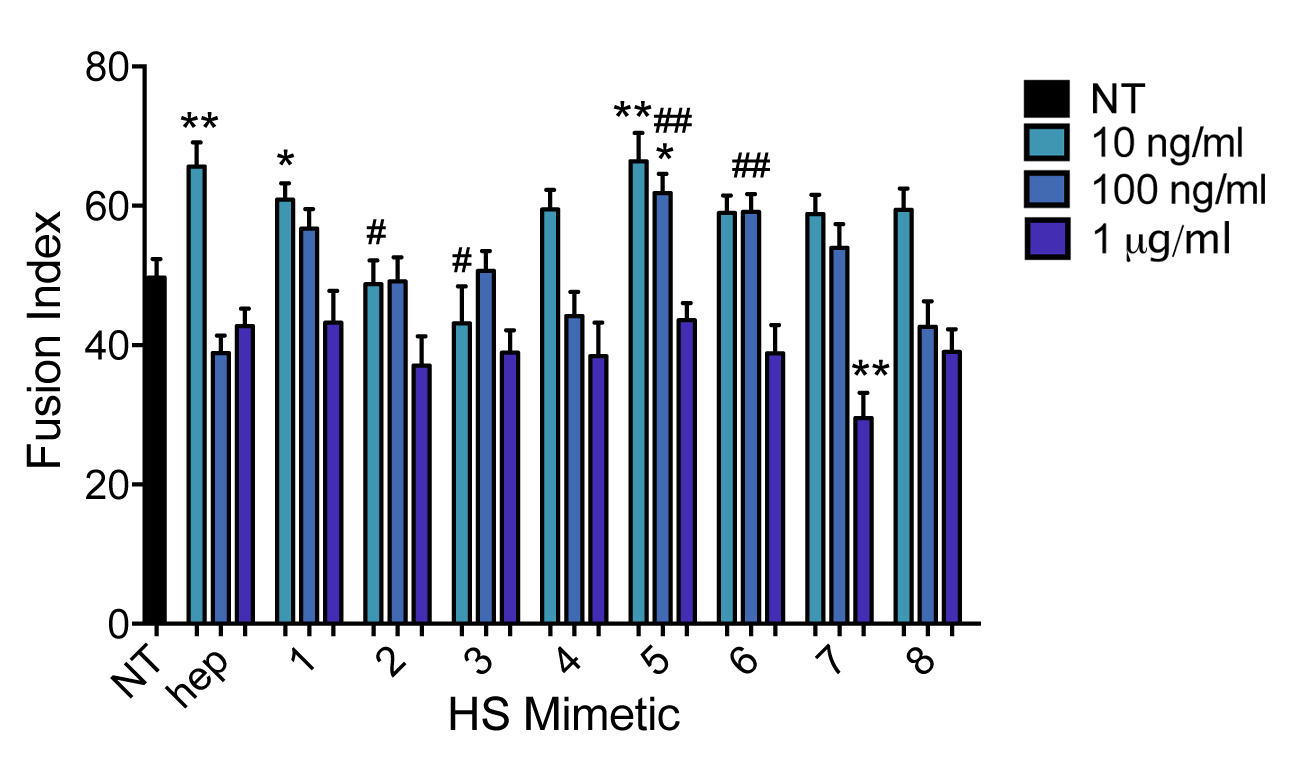


**Figure S2: Myoblast fusion is affected by different heparan sulfate mimetics in a similar way to myoblast differentiation.** Primary satellite cell-derived myoblasts were induced to differentiate for three days by serum withdrawal prior to fixation and immunostaining to detect myosin heavy chain (MyHC, green) and DNA (DAPI, blue). The fusion index was calculated as the percentage of nuclei contained in MyHC+ multinucleated cells over the total number of nuclei contained in all MyHC+ cells (both mono- and multi-nucleated). The fusion index follows a similar trend to that observed for the differentiation index (Figure 3) and thus, since differentiation precedes fusion. These results suggest that myoblast fusion is not significantly affected by changes in the extracellular heparanome. Results from 10 random fields across two technical replicates and three independent biological replicates were averaged and plotted ± S.E.M. Asterisks are p-values for each condition compared to non-treated (NT) cells, while hash signs are p-values for each condition compared to heparin. *=p<0.05, **=p<0.01, #=p<0.05, ##=p<0.01.
